# Artificial Intelligence in Fetal and Pediatric Echocardiography

**DOI:** 10.3390/children12010014

**Published:** 2024-12-25

**Authors:** Alan Wang, Tam T. Doan, Charitha Reddy, Pei-Ni Jone

**Affiliations:** 1 Division of Pediatric Cardiology, Ann & Robert H. Lurie Children’s Hospital of Chicago, Chicago, IL 60611, USA; pejone@luriechildrens.org; 2 Division of Pediatric Cardiology, Department of Pediatrics, Texas Children’s Hospital, Baylor College of Medicine, Houston, TX 77030, USA; tam.doan@bcm.edu; 3 Division of Pediatric Cardiology, Stanford Children’s Hospital, Palo Alto, CA 94304, USA; reddyc@stanford.edu

**Keywords:** artificial intelligence, pediatric echocardiography, fetal echocardiography, congenital heart disease, machine learning, deep learning

## Abstract

Echocardiography is the main modality in diagnosing acquired and congenital heart disease (CHD) in fetal and pediatric patients. However, operator variability, complex image interpretation, and lack of experienced sonographers and cardiologists in certain regions are the main limitations existing in fetal and pediatric echocardiography. Advances in artificial intelligence (AI), including machine learning (ML) and deep learning (DL), offer significant potential to overcome these challenges by automating image acquisition, image segmentation, CHD detection, and measurements. Despite these promising advancements, challenges such as small number of datasets, algorithm transparency, physician comfort with AI, and accessibility must be addressed to fully integrate AI into practice. This review highlights AI’s current applications, challenges, and future directions in fetal and pediatric echocardiography.

## 1. Introduction

Echocardiography is an essential diagnostic tool used to assess cardiac structures and function in children. While it provides real-time, non-invasive imaging, the interpretation of echocardiographic data can be complex and requires significant expertise. Availability of resources, variability in operator skill, and the subtlety of certain abnormalities can lead to diagnostic challenges and disparities in access to care.

In recent years, advancements in artificial intelligence (AI) have begun to revolutionize many areas of healthcare, including diagnostic imaging. AI refers to any technique that enables computers to generate algorithms, find hidden insights, and mimic human learning and thinking [1,2]. Machine learning (ML) is a subfield of AI that requires input of data to allow a computer to learn and perform a task without explicit programming. Deep learning (DL) is a subset of ML that mimics the human brain by using artificial neural networks (ANNs) to analyze large datasets and draw conclusions in a human-like manner. Machine learning algorithms, particularly those involving DL, can be trained to recognize patterns in echocardiographic images and improve accuracy in detecting cardiac anomalies and quantifying cardiac function. However, it can be time-consuming and challenging to develop and process large image datasets as each image needs to be labeled, stored, and DL models require powerful computational resources [1]. AI applications in fetal and pediatric echocardiography are still in the early stages, but show promise for improving diagnostic accuracy, increasing access to care, facilitating early detection of congenital heart disease (CHD), and predicting outcomes.

This review explores the current applications of AI in fetal and pediatric echocardiography. Our literature search was completed on PubMed with search queries of “artificial intelligence” or “machine learning” and “fetal echocardiography” or “pediatric echocardiography”. The literature search only included published articles in the English language. By examining the latest research and clinical implications, this article aims to provide a comprehensive and up-to-date understanding of how these technologies are shaping the field and the benefits they may offer in improving pediatric cardiac care.

## 2. Basic Concepts of AI in Echocardiography

Artificial intelligence ML and DL algorithms aim to autonomously perform programmed tasks or find hidden insights within complex sets of medical data. These algorithms can be divided into three main categories: supervised learning, unsupervised learning, and reinforcement learning [2,3,4,5,6]. Supervised learning algorithms are trained on data that are labeled with the outcome to “learn” [7]. For example, a dataset of images labeled as “normal” or “congenital heart disease” would be the input, and the algorithm learns to associate image features with these labels and subsequently identifies the outcome in new, unseen images. This method is useful for tasks such as disease classification, quantifying cardiac function, and identifying cardiac structures. Unsupervised learning algorithms work with data that lack labeled outcomes [5]. This method is used to uncover hidden patterns or groupings within the data. In echocardiography, unsupervised learning can help identify previously unrecognized patterns in cardiac morphology or function. Reinforcement learning is an algorithm that learns through trial and error [6] and has the potential for optimizing decision making, such as guiding treatment strategies based on real-time data during an echocardiogram [8]. Reinforcement learning algorithms in cardiology are scarce and are especially difficult to establish in pediatric echocardiography due to lack of large, labeled datasets that are essential for training. Factors such as high anatomic variation and noise in pediatric echocardiographic images further complicates reinforcement algorithm development.

Deep learning has transformed the field of medical imaging by enabling computers to automatically learn from vast amounts of data through supervised and unsupervised tasks. One of the most powerful DL techniques used in image analysis is the convolutional neural network (CNN) [6]. CNNs are designed to mimic the way the human brain processes visual information by consisting of multiple layers of interconnected neurons that perform different operations. CNNs can learn complex and subtle features from images and fine-tune predictions during training. The remarkable capabilities of ML have been facilitated by several key develops in the AI field over the past decade and include: (1) the development of hardware such as graphical processing units that can perform massive amounts of calculations simultaneously; (2) the increasing availability of datasets for training AI systems; and (3) the application of complex AI algorithms like neural networks [9,10].

**Figure 1:**
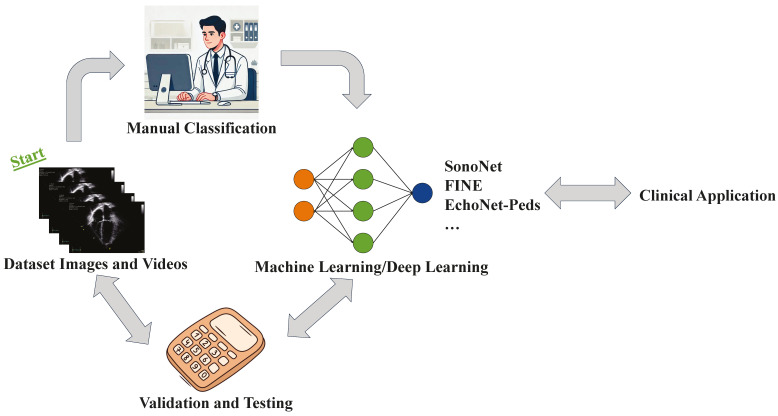
Flowchart of Designing, Testing, and Validating a Machine Learning Algorithm in Pediatric Echocardiography. The process starts with collecting a large dataset of images and/or videos. Manual classification of each image view and the individual structures within those views is performed. The classified images are uploaded to a machine learning algorithm for learning. The algorithm is then validated, tested, and applied clinically. Additional images and continual feedback are used for further re-training and refinement of the algorithm.

The process of designing, training, and validating an AI algorithm for the detection of fetal or pediatric congenital heart disease is complex and labor intensive. Currently, the process starts by collecting a large dataset of normal and abnormal images and/or videos and manually labeling features of interest. After the dataset is labeled and low-quality images are removed, an appropriate ML algorithm is chosen. The images are then split into a training, validation, and test set and the training dataset is applied to create the model. The model is then evaluated on the validation model and optimized. If the model performed well, it may be further refined and integrated into clinical workflow. Further refinement of the algorithm may occur through feedback and re-training with additional images (Figure 1).

Fetal and pediatric echocardiography present unique challenges compared to adult echocardiography due to variability in heart size, heterogeneity of CHD, and difficulty in obtaining high-quality images because of both patient and respiratory movement or surgical artifact [2,11]. AI algorithms may be trained to overcome these challenges by automating view classification, function analysis, and detection and analysis of CHD. In doing so, it may reduce reliance on human interpretation, which can be subjective and operator dependent. Automation of specialized tasks may be particularly valuable in resource-limited settings where pediatric cardiology expertise is scarce.

## 3. Current Applications of Artificial Intelligence in Fetal Echocardiography

Congenital heart disease is the most common cause of birth defect, with a prevalence of 6 to 12 cases per 1000 live births [12,13]. Prenatal screening for CHD can improve neonatal outcomes and offer guidance on pre-and post-natal interventions [14,15,16]. Fetal ultrasound screening is recommended at 18 to 22 weeks gestation for every pregnancy at risk for CHD [17]. The prenatal CHD diagnostic rate, however, is only 30–50% in community obstetrician/gynecology practices [13,18,19,20,21]. Despite being the most common cause of birth defects, the prevalence in the population is still low and increases the chance of non-specialized reviewers overlooking or not recognizing abnormalities. AI may be able to bridge this gap in expertise with automated detection of CHD, retrieval of standardized planes, and identification of cardiac structures. In this section, we will further discuss recent applications of AI in fetal echocardiography (Table 1).

### 3.1. Image Acquisition, Classification, and Optimization

The difficulties in prenatal diagnosis of CHD are attributed to anatomic complexity of the heart, small patient size, and fetal motion. The initial step in a diagnostic fetal echocardiogram is obtaining the standard echocardiographic views, which can be time consuming and requires extensive expertise and skill. Prior studies have used CNNs such as SonoNet to automatically detect standard fetal views, including some cardiac views, in real-time with >90% accuracy [26,27]. This reduced the time for manual annotation and negated the need for a sonographer to stop and obtain necessary images [11,28].

The acquisition of fetal cardiac images has been made easier with the introduction of a 4D sonography technology called spatiotemporal image correlation (STIC). STIC allows the acquisition of a fetal cardiac volume dataset and displays a cine loop of a single complete cardiac cycle [29,30,31]. Manual navigation of STIC volumes may be used to examine the normal fetal heart, but requires a comprehensive understanding of fetal anatomy and is highly operator dependent [32,33]. Recently, a novel method called Fetal Intelligent Navigation Echocardiography (FINE) applied intelligent navigation technology to STIC datasets to automatically detect and display nine standard fetal echocardiography views [25]. Using STIC, Yeo and colleagues obtained 4D volume datasets of the fetal heart from the apical four-chamber view by transverse sweeps [25]. FINE was trained to interrogate the STIC volume dataset and automatically generate and display the nine standard fetal views so that manual manipulation was no longer needed. On testing, FINE was able to generate the nine fetal echocardiography views using diagnostic planes in 78–100% of the cases. The highest detected planes were the four chamber, five chamber, and abdomen/stomach views (92%, 92%, and 100%, respectively), and the lowest detected planes were the ductal arch and SVC/IVC plane (73% and 80%, respectively). Since the initial publication and introduction of FINE in 2013, it has been continually refined and is now integrated into multiple ultrasound platforms like 5D Heart [34]. FINE has been demonstrated in recent studies to obtain standardized fetal echocardiography views in >92% of scans performed by non-expert sonographers after a short training period of 1–2 h [34,35]. FINE also significantly reduced the mean time to obtain nine cardiac views from approximately 16 min to just over 2 min [34].

Deep learning can be utilized for segmentation and automatic detection of cardiac substructures. Segmentation of cardiac structures is an important step in image analysis, as it subdivides the images into regions such as ventricles, atria, and great arteries. Automating this step could improve morphologic assessment and accuracy in measurements [36]. DW-Net was developed to segment the fetal heart in the apical 4-chamber view. DW-Net is a combination of a dilated convolutional chain which localizes cardiac chambers and a W-Net that aids in detecting precise boundaries and performs segmentation. After training with a dataset of 895 images, the model was able to identify cardiac structures such as the atria, ventricles, and descending aorta with an accuracy of ~80% [37]. Komatsu et al. used DL to create a novel architecture called Supervised Object detection with Normal data Only (SONO) for the purpose of detecting cardiac substructures [23]. The authors trained the model by manually annotating the correct positions of 18 different anatomical substructures from 247 normal fetal ultrasounds. Cardiac structures such as the ventricular septum, both ventricles, and atria were better detected (average precision > 0.7 in the testing cohort). Detection of the tricuspid valve, mitral valve, inferior vena cava, pulmonary veins, and ductus arteriosus were poor (average precision < 0.5 in testing cohort) and may have been attributed to their small size. To test the detection of abnormal structures in the heart and vessels, SONO analyzed videos of 14 CHD cases. ROC analysis showed that there was an area under the curve (AUC) of 0.787 for detecting abnormalities in the heart and 0.891 in the vessels. Artificial intelligence has also been employed to automatically collect non-image data such as biometric measurements and produce reports. When implemented, these tools saved 7.6 min per scan and may help sonographers concentrate on image interpretation by removing disruptive tasks [38].

### 3.2. Detection of Congenital Heart Disease

Artificial Intelligence can be used as a decision support tool to detect and diagnose CHD in fetal echocardiography. For example, Truong et al. constructed random forest (RF) models to differentiate patients with CHD [24]. The authors used 25 features to train a baseline RF model and then selected 10 top features for the final training and optimization of the RF model for CHD prediction. The study population was derived from a database of 3910 singleton fetuses. The proportion of CHD was 14.1%, confirmed by post-natal echocardiograms. The AUC for detection of CHD was 0.94, with a sensitivity of 0.85 and specificity of 0.88. The model was shown to have similar performance in detecting CHD with 6 features compared to 10. The six most important features were cardiac axis, peak velocity of blood flow across the pulmonary valve, cardiothoracic ratio, pulmonary and aortic valve annular diameter, and right ventricular end-diastolic diameter. The sensitivity of 0.85 and negative predictive value of 97% in this study shows that ML may have the potential to improve prenatal CHD screening by alerting the echocardiographer and interpreting physician to the potential presence of CHD.

Deep learning has also been applied to detection of CHD in fetal echocardiography. Arnaout et al. implemented an ensemble of neural networks to identify five diagnostic-quality cardiac views, provide classification of normal anatomy versus 16 complex CHD lesions, and calculate fractional area change for each cardiac chamber [22]. The five cardiac views included the 3-vessel trachea, 3 vessel, left ventricular outflow tract, apical 4 chamber, and abdominal views. The authors trained the CNN with 107,823 images from 1326 screening fetal echocardiograms. The CNN had an AUC of 0.89–0.99 in distinguishing normal from abnormal hearts with a sensitivity of 95%, specificity of 96%, and negative predictive value of 100%. The performance of their model was comparable to clinicians who reviewed the same collection of images in sensitivity and superior in specificity. To ensure that the model would work in the real world, the CNN was trained using 2D ultrasound and standard recommended fetal views rather than specialized or vendor-specific image acquisitions. This model was later tested in a community setting and achieved a sensitivity and specificity of 91% and 78%, respectively—a significant achievement in a cohort in which over 50% of CHD cases were initially missed clinically [39]. Thus, this model could be helpful in resource limited countries to help detect CHD.

Artificial intelligence programs in fetal echocardiography have the potential to enhance image acquisition, classification, and optimization. Programs such as SonoNet and FINE can significantly aid sonographers and clinicians by improving efficiency and accuracy. Deep learning models like DW-Net and SONO can aid in precise morphologic assessments by detecting and segmenting fetal cardiac structures. Although these models achieve moderate to high accuracy for some cardiac structures, detection remains challenging for smaller or more complex anatomical features. A more immediate implementation of AI may be its use in streamlining workflow by automating tasks such as collecting biometric measurements during scanning. When clinically ready, these AI programs have the potential to serve as valuable clinical decision support tools, especially in resource limited settings, and may improve fetal CHD detection and screening.

## 4. Current Applications of Artificial Intelligence in Pediatric Echocardiography

**Figure 2:**
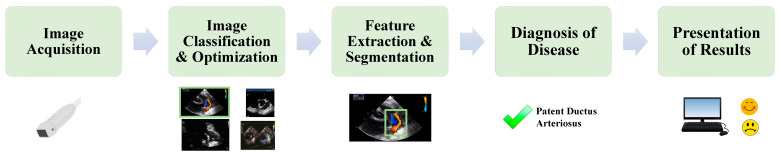
AI Optimization of the Echocardiographic Imaging Pipeline: Detection of a PDA. After an image is acquired, AI may assist in the detection of a PDA through automated image optimization and view classification. Upon finding the typical views of detecting a PDA, the algorithm searches the image(s) to detect features consistent with a PDA. Based on findings during feature detection, the algorithm will determine whether a PDA is present. Those results will be displayed for the clinician and feedback may be provided to the algorithm for reinforcement learning. At present, most AI algorithms are not able to autonomously complete the entire imaging pipeline.

Pediatric heart disease is a leading cause of morbidity and mortality worldwide [40]. Transthoracic echocardiography is the primary imaging modality for the diagnosis and surveillance of pediatric acquired and CHD. Like fetal echocardiography, image acquisition and analysis are operator-dependent and requires extensive training and experience, especially in the setting of CHD [41]. Interpretation of transthoracic echocardiography is also time consuming, as most studies have > 100 images. Accurate measurements of heart size and function are crucial in accurate diagnosis, but often have wide limits of interobserver variability [42]. The application of AI in pediatric echocardiography has potential to improve multiple aspects of the imaging pipeline, including image acquisition, image optimization, automating measurements, disease identification, and decision support (Figure 2). In the following section, we will discuss recent applications of AI in pediatric echocardiography (Table 2).

### 4.1. Image Acquisition, Classification, Optimization, and Automated Measurements

If a major goal for AI in pediatric echocardiography is to autonomously diagnose CHD from a standardized scan, one of the first steps is for AI to assist with image acquisition and autonomously classify standard views. View classification is an important step, as it enables the development of models to aid acquisition, optimization, and interpretation [54]. Østvik et al. trained a CNN to automatically identify seven standard echocardiography views in adult patients and help guide sonographers to obtain optimal angles [55]. Automatic view classification has also been developed for pediatric echocardiograms [46]. Gearhart et al. built an AI model using 642 echocardiograms from unique patients encompassing 27,948 clips for training, validation, and testing. A human trainer labeled twenty-seven preselected views of interest for each echocardiogram. The preselected views are all part of the standard pediatric echocardiography protocol set by the American Society of Echocardiography [56] and were chosen to include views from varying transducer locations.

The overall accuracy of the testing cohort was 90.3% and did not differ based on age group. The model accuracy was slightly higher in patients with normal hearts versus those with CHD (91.4% versus 88.2%, *p* < 0.001) [46]. The views with the highest accuracy (>91%) included the apical 2-chamber, 3-chamber, and 4 chamber sweeps, apical 4-chamber mitral valve pulse wave doppler, and subcostal descending aorta pulse wave doppler. The views with the lowest accuracy (<72%) included the subcostal short-axis sweep, high left parasternal transverse branch pulmonary arteries, parasternal short axis papillary muscle, parasternal long axis mitral valve, and parasternal short-axis aortic valve. The algorithm often confused similar appearing views with each other. This model did not include patients with complex CHD, as a larger sample size was needed.

View classification has also been investigated by Wu et al., who built a classification model to identify 23 standard pediatric echocardiography views [52]. Their study trained an AI algorithm with 3409 echocardiograms encompassing 247,750 images. The echocardiograms included a mix of normal hearts, unrepaired CHD, and CHD after surgery. Their model was able to correctly classify views with a majority of F1 scores ≥ 0.90. Accurate segmentation of cardiac chambers is crucial for accessing chamber dilation and ejection fraction. Both pediatric and adult studies have shown that AI models can obtain chamber size and ejection fraction values congruent with an expert performing manual segmentation [54,57].

Left ventricular ejection fraction (LVEF) is a commonly used modality to quantify cardiac function. LVEF assessment by echocardiography can be associated with a wide range of interobserver variability based on operator experience [58,59]. Automated calculation of LVEF may improve accuracy and reduce interobserver variability. Reddy et al. and Zuercher et al. both independently retrained an adult ML algorithm, EchoNet-Dynamic, to fully automate assessment of left ventricular function [50,53]. EchoNet-Dynamic, was previously trained using 10,030 adult echocardiograms with associated LVEF values [60]. Reddy et al.’s model, named EchoNet-Peds, employed a sequential segmentation and ejection fraction estimation task. Their algorithm estimated ejection fraction with a mean absolute error of 3.66% and had an AUC of 0.954 for diagnosing echocardiograms with an ejection fraction < 55% [50]. This model was also able to provide a visual representation of the segmentation task to demonstrate how ejection fraction was calculated. Zuercher et al. retrained EchoNet-Dynamic with 240 echocardiograms from unique pediatric patients. The calculated LVEF in the retrained testing group had a mean absolute error of 4.47% and mean bias of −2.42% when compared to the manual tracings of expert echocardiographers. When Zuercher and colleagues analyzed the same testing group with the untrained adult EchoNet-Dynamic model, it produced a higher mean absolute error of 8.39%. These studies demonstrate that existing adult echocardiography algorithms could be retrained with a relatively small amount of pediatric data and yield significantly more accurate results. Further, the results suggests that features learned from adult data transferred well to pediatric data and leveraging more available adult data may be helpful in training future pediatric models [53].

The same EchoNet-Dynamic DL algorithm has been also retrained on a separate set of pediatric echocardiograms to calculate cardiac output [51]. Ufkes et al. modified the DL model to calculate left ventricular outflow tract diameter from the parasternal long axis view in pediatric patients [51]. They then developed a DL approach to estimate velocity time integral from the left ventricular outflow tract and used the combined model to estimate cardiac output. The calculated cardiac index had a root mean absolute error of 0.398 L/min/m^2^, R^2^ of 0.755, and mean bias of 0.14 L/min/m^2^ compared to an expert reviewer. The left ventricular outflow tract diameter and velocity time integral were predicted with a mean bias of 0.32 mm and 0.20 cm, respectively. Notably, the mean bias of the velocity time integral between two expert reviewers was 0.49 cm, significantly higher than the bias between the algorithm and primary reviewer. AI algorithms such as AutoLV and AutoSTRAIN (TomTec) for automating the assessment of LV function have already been employed in commercial workstations. These features have proven feasible in normal pediatric hearts and produce results that are comparable to traditional methods in less time [61]. The automated view classification and measurements have the potential to reduce the amount of time spent on manual measurements. The amount of time saved can allow pediatric cardiologists to focus on complex CHD rather than on normal echocardiograms.

### 4.2. Detection and Diagnosis of Congenital Heart Disease

Unlike computed tomography (CT) or magnetic resonance imaging (MRI), the selection of pertinent views in echocardiography can be subjective and varies based on image quality. The selection and standardization of echocardiography views is crucial for the application of DL in detecting CHD. Jiang et al. developed a method to detect CHD using seven standard echocardiography views (parasternal long axis, parasternal short axis aortic valve, parasternal 4 chamber, parasternal 5 chamber, subxiphoid 4 chamber, subxiphoid biatrial view, and suprasternal long axis view). After training with labeled data by expert reviewers, the AUC of detecting CHD was 0.91 and accuracy was 92.3% [47]. This DL model only detected the presence of CHD but was not able to discriminate types of CHD.

Over the past few years, algorithms have been developed to help detect common CHDs such as atrial septal defects (ASD) and ventricular septal defects (VSD) with good accuracy [48,62]. Lin et al. developed and validated a DL model for automated detection and quantification of ASDs [48]. Their algorithm was unique in that it both provided view classification and diagnosis. The DL model was trained to identify four standard color views commonly used in the diagnosis of ASD with an average accuracy of 0.99. The composite AUC for diagnosing ASD was 0.92 with a sensitivity of 87.8% and specificity of 89.4%. The algorithm was also trained to automatically calculate ASD defect size and septal length. The mean bias of 1.9 mm and 2.2 mm for defect size and total septal length was similar to that of the expert reviewers. Additionally unique to this study was the ability of the algorithm to provide keyframes used for detection of the ASD and measurements of the ASD and septal length. Since a major limitation with DL models is the lack of transparency in AI data interpretation e.g., the “black box” conundrum, visual confirmation of diagnostic images may improve trust during utilization. Additional CNNs have been previously developed to differentiate transposition of the great arteries from normal using adult data [63]; however, their performance with neonatal and pediatric images has not yet been evaluated.

Artificial intelligence has proved helpful in exploring novel associations within big data using unsupervised cluster analysis. This modality was used by the Congenital Heart Surgeons’ Society (CHSS) to identify distinct, novel, and clinically relevant groups of patients with critical left heart obstruction (CLHO) using baseline quantitative and qualitative measurements [64]. In this study, 651 pre-intervention transthoracic echocardiograms, each with 136 separate measures, were analyzed through unstructured cluster analysis. Cluster analysis allowed for the grouping of similar patients based on the structure of echocardiographic data, each variable was given equal weight, and the number of clusters was not pre-determined. Cluster analysis identified 3 distinct groups, each with at least 98 patients. The most important distinguishing features between the three groups, via multinominal regression, were left ventricular end diastolic area and degree of aortic valve atresia. Prior consensus statements had dozens of diagnostic categories for CLHO, such as mitral valve characteristics and pulmonary vein abnormalities, and cluster analysis showed a much simpler relationship [65,66].

Machine learning can also assess specific features in echocardiography such as pulmonary regurgitation (PR). Cohen et al. created a cross-validated multivariable model to predict cardiac magnetic resonance imaging derived pulmonary regurgitation fraction from echocardiographic and demographic variables [44]. They included the following echocardiographic variables as predictors: PR index, PR pressure half time, main pulmonary artery flow reversal, vena contracta ratio, and type of pulmonary outflow tract at time of imaging. Patients who underwent cardiac magnetic resonance imaging and echocardiography within 3 months of each other were included in the study. Their model was able to distinguish mild from greater than mild PR with excellent discrimination (AUC 0.96) and performed similarly to trained clinicians. Notably, the group found that the multivariable algorithm did not perform any better than a simple univariable model which incorporated the presence of branch pulmonary artery flow reversal. This study further highlights the ability of AI to take a collection of complex variables to find hidden, and sometimes, simple associations.

### 4.3. Use of Artificial Intelligence in Pediatric Echocardiography to Predict Patient Outcomes

Cluster analysis has also been employed to find hidden associations between echocardiographic variables, clinical characteristics, and medical treatment of patients with dilated cardiomyopathy [67]. Using a cohort of 47 patients with dilated cardiomyopathy and 25 healthy children, Garcia-Canadilla et al. performed an unsupervised ML cluster analysis that yielded five groups, with the first two groups containing controls and groups three to five containing patients with dilated cardiomyopathy. Further analysis of the dilated cardiomyopathy groups exhibited that a prolonged isovolumic contraction time and decreased and delayed outflow velocity was seen in the high-risk groups. Patients with more systolic dysfunction were more frequently clustered in a group with higher adverse outcomes while patients with predominantly diastolic dysfunction were mostly found in a lower risk group. These studies highlight the ability of unbiased clustering to identify new phenotypes and provide new evidence to help clinicians with prognostication and risk stratification.

### 4.4. Screening for Acquired Heart Disease

Rheumatic heart disease (RHD) is a significant cause of morbidity and mortality in low-and middle-income countries [68]. RHD is a progressive disease that often presents late, with severe valvar dysfunction and heart failure [69]. Screening echocardiography can detect RHD during the latent period and enable early initiation of antibiotics, which reduces the risk of disease progression [70]. The implementation of screening echocardiograms in low-and middle-income countries is a challenge due to the lack of infrastructure, equipment, and trained sonographers and cardiologists. AI programs that can assist in image acquisition and interpretation may decrease the burden for trained experts.

Screening for RHD using AI-guided echocardiography in image acquisition and interpretation has been recently evaluated. Brown et al. trained a ML algorithm to detect mitral regurgitation (MR) and perform MR jet analysis for detection of RHD [43]. The model’s discrimination between RHD and non-RHD had an AUC of 0.93, precision of 0.83, recall of 0.92, and F1 of 0.87. The average length of the MR jet via ML closely aligned with manual measurements by cardiologists (*p* = 0.83). The detection of MR in pediatric patients has also been examined by Edwards et al., who developed a fully automatic ML software capable of view classification and MR detection [45]. This software correctly classified the parasternal long axis color view with an accuracy of 0.98 and F1 of 0.97. The detection of MR had an AUC of 0.91. Of note, both studies developed and tested ML algorithms on portable echocardiography machines, which may limit accessibility in countries where RHD is endemic.

A separate group, Peck et al., trained novices to produce diagnostic quality images for RHD using handheld echocardiograms. Novices received 5 h of training with a handheld echocardiogram machine (uSmart 3200t Ultrasound System [Terason]). Navigational Guidance (Caption Health) was integrated on the handheld system and provided AI guidance to obtain diagnostic views [49]. After training, novices were able to obtain diagnostic images over 80% of the time in the parasternal long axis black and white and color views. The diagnostic quality of the apical 5-chamber view was poor, at less than 50%. Cumulatively, however, novice sonographers were able to obtain diagnostic images for 91% of patients. These studies highlight the use of AI to help screen for RHD and will be helpful in resource limited countries where RHD is quite prevalent. Uses of AI in other acquired heart disease will need to be explored in the future.

Artificial intelligence has the potential to revolutionize pediatric echocardiography by streamlining image acquisition, optimizing measurements, and enhancing disease detection. For example, CNN-based algorithms have demonstrated high accuracy in view classification and may help sonographers in obtaining optimal images [46,52]. Additionally, AI models such as EchoNet-Peds have automated the calculation of left ventricular ejection fraction, reducing interobserver variability and allowing for more efficient workflows [50,53]. By guiding image acquisition and providing automated analysis, AI can reduce the workload on clinicians, enable faster and more accurate diagnostics, and improve access to care (Table 3).

## 5. Challenges of Artificial Intelligence in Fetal and Pediatric Echocardiography

This paper reviewed the ways that AI has been successfully utilized to assist with image optimization, processing, and interpretation in fetal and pediatric echocardiography. There are, however, significant challenges in implementing AI in fetal and pediatric echocardiography at the bedside. These challenges include small datasets, lack of explainability in algorithms, physician comfort level with AI, ease and availability of access, and ethical and legal concerns.

One of the significant challenges in applying AI and ML to fetal and pediatric echocardiography is the relatively small size of available databases. Unlike in adult cardiology where there are vast amounts of echocardiographic data, fetal and pediatric cases are rarer, especially for CHD. The low volume of data can hinder training and validation of AI algorithms such as CNNs, which may require thousands of annotated images to make reliable predictions. Algorithms that rely on single-center data may produce inherent bias based on the unique interconnection of patient demographics, types of CHD lesions, and outcomes. This raises ethical concerns, as datasets which are not representative of the boarder fetal and pediatric population may inadvertently favor certain patient groups while disadvantaging others [71]. A large barrier to multicenter collaboration is that, while individual centers may have large databases of patient images, there is currently no standardized method for building, sharing, and ensuring patient confidentiality in large DL databases [28]. Currently, many AI studies lack external validation, which limits physician buy-in [2]. One way of overcoming low database volume in pediatrics is through transfer learning. In transfer learning, AI models that are trained on large adult echocardiography datasets are fine-tuned using smaller pediatric datasets. The retraining of EchoNet-Dynamic for pediatric echocardiograms is an example of how transfer learning takes advantage of previously learned features from adult data and adapts to specific characteristics of pediatric echocardiography. Public sharing of de-identified fetal and pediatric images will help in future collaborative efforts. The Stanford Medicine team have made their dataset of 4467 pediatric echocardiograms publicly available [50].

A second challenge in implementing AI in pediatric echocardiography is the “black box” nature of many ML models, particularly DL algorithms such as CNNs. While these algorithms may achieve similar accuracy as trained experts, the lack of transparency yields significant concerns in fields such as pediatric cardiology, where trust and accuracy are paramount. Black-box algorithms pose challenges when errors occur, as it is difficult to pinpoint why or how the algorithm made the error. This lack of interpretability makes it harder to identify biases, shortcomings in training data, or specific areas where the model can be improved. Furthermore, clinicians are responsible for explaining clinical decisions to patients and families. Artificial intelligence complicates the counseling process, as there may not be an easy way to explain AI’s reasoning, leading to a lack of trust between patients and providers [71]. Programmers have started to partially solve the challenges posed by opaque AI models by highlighting locations on images where decisions and/or measurements are made with saliency maps [22,52,72] or key frames [48,50].

Since AI is a nascent field, especially in pediatric cardiology, there is a lack of adequate physician knowledge and understanding of AI technologies. This knowledge gap creates hesitation and skepticism about integrating AI tools into clinical practice. Addressing this knowledge gap will require integrating AI education into medical training programs and continuing medical education (CME) for practicing physicians. Targeted CME programs can offer workshops and case-based learning opportunities for sonographers and pediatric cardiologists. Due, in part, to a lack of transparency and ethical and legal concerns, it is unlikely that AI will exist in clinical practice without physician oversight in the foreseeable future [73]. Therefore, a clinician’s ability to critically appraise AI models is needed for integration. Additionally, interdisciplinary collaboration between physicians, data scientists, and AI developers is essential in ensuring that AI is developed in a manner that is user-friendly, clinically relevant, and addresses inherent biases.

Lastly, while AI can streamline workflows and deliver expert-level diagnostics in underserved regions, many of the current AI tools are only in the experimental phase and not yet integrated into commercial portable or handheld echocardiogram machines. For AI to be effective in pediatric echocardiography, it must be seamlessly integrated into clinical practice and allow physicians and sonographers to use the tools without disruption to their workflow. If AI systems require clinicians to undergo extensive training or require complex post-processing steps and calibration, they could add time to clinical workflows and are thus unlikely to be adopted widely. Accessibility is further complicated by variability in healthcare infrastructure across different regions and institutions. In high-resource settings, technical support teams may be available to help clinicians troubleshoot and better integrate AI into the clinical setting. However, in low-resource settings, AI tools may be limited by a lack of support infrastructure, internet connectivity, storage, and computing abilities. Notably, certain medical conditions that could potentially benefit from AI, such as RHD, are endemic in low-resource regions. Thus, while AI has the potential to revolutionize how medicine and clinical care is delivered, it may also further widen the disparity in healthcare equity.

## 6. Future Directions

A multidisciplinary approach is needed to address the multitude of challenges in implementing AI in fetal and pediatric echocardiography. A 2022 review article on AI in congenital heart disease published in the Journal of the American College of Cardiology identified four key areas to focus on in CHD: (1) rare diseases such as coronary artery anomalies; (2) acquired diseases that disproportionately affect patients with limited access, such as RHD; (3) CHDs with high morbidity and mortality such as failing Fontan; and (4) precision medicine for decision-making in difficult diseases such as borderline left heart [2]. Although this article was addressing AI in CHD, these key areas are already topics of prior research and current interest in pediatric echocardiography. Regardless of current trends in AI research, collaboration between multiple disciplines and institutions is critical in future design and implementation.

## 7. Conclusions

The integration of AI into fetal and pediatric echocardiography has the potential to improve diagnostic accuracy, enhance workflow efficiency, increase access to care, and improve patient outcomes. While there are still many challenges to overcome before AI is commonplace in the pediatric echocardiography laboratory, ongoing advancements in AI technology, data sharing, and interdisciplinary collaboration are helping to remove barriers. However, continued research and development of more transparent, accessible, equitable, and user-friendly AI tools are needed to fully realize the benefits of AI in pediatric echocardiography.

## Figures and Tables

**Table 1:** Summary of Select AI in Fetal Echocardiography Articles.

Paper	Objective	Technology	Sample Size	Performance
Arnaout et al. [22]	CHD detection	Deep learning	107,833 images from 1326 studies	AUC 0.99, 95% sensitivity, 96% specificity, and 100% negative predictive value for distinguishing normal from abnormal hearts
Komatsu et al. [23]	Image segmentation	Deep learning	8182 frames from 247 fetal studies	AUC of 0.787 in the heart and 0.891 in the vessels for detecting cardiac substructures
Truong et al. [24]	CHD detection	Machine learning	3910 fetal echo examinations	ROC of 0.94, sensitivity 85%, specificity 88%, and negative predictive value 97% for detection of CHD
Yeo et al. [25]	Image acquisition & classification	FINE	51 volume datasets	Generates 9 fetal echo views using diagnostic places in 98–100% of cases

**Table 2:** Summary of Select AI in Pediatric Echocardiography Articles.

Paper	Objective	Technology	Sample Size	Performance
Brown et al. [43]	Screening for acquired heart disease	Machine learning	282 rheumatic heart disease and 229 normal echos	AUC of 0.93, precision of 0.83, and recall of 0.92 of discriminating rheumatic heart disease and normal
Cohen et al. [44]	Disease specific diagnosis	Machine learning	243 patients	AUC of 0.96 for prediction of >mild pulmonary regurgitation
Edwards et al. [45]	Disease specific diagnosis	Machine learning—convolutional neural network	66,330 frames for development and 11,730 frames for testing	ROC 0.91 for detection of mitral regurgitation
Gearhart et al. [46]	View classification	Machine learning—convolutional neural network	642 echocardiograms for training, validation, and testing. 524 echocardiograms in children with leukemia for practical application.	Accuracy was 90.3% for 27 preselected views. For leukemia patients, PPV of 98.7% to 99.2% and sensitivity of 76.9% to 94.8% for 6 preselected views.
Jiang et al. [47]	View classification and screening for congenital heart disease	Deep learning	1411 echocardiograms from which 7 views were selected	AUC of 0.91 and accuracy of 92.3 for detection of congenital heart disease
Lin et al. [48]	Disease specific diagnosis	Deep learning	48 atrial septal defect echocardiograms and 377 echocardiograms without atrial septal defect	ROC for atrial septal defect was 0.92. Segmentation had mean bias of 1.9 mm and 2.2 mm for defect size and total septal length, respectively compared to expert
Peck et al. [49]	Disease specific diagnosis	Machine learning	462 echocardiograms (362 by non-experts with AI guidance and 100 by experts without AI guidance)	Novice images enables diagnostic interpretation in > 90% of studies for presence or absence of rheumatic heart disease
Reddy et al. [50]	Automated function quantification	Deep learning—EchoNet-Peds	4467 echocardiograms from 1958 patients	Mean average error for ejection fraction was 3.66%. ROC of 0.954 for diagnosing echocardiograms with left ventricular ejection fraction < 55%.
Ufkes et al. [51]	Automated function quantification	Deep learning—EchoNet-Dynamic	260 echocardiograms	Cardiac index calculated with mean standard error 0.389 L/min/m^2^.
Wu et al. [52]	View classification	Deep learning	367,571 echocardiographic image slices from 3772 patients	Many views required to diagnose septal defects, pulmonary stenosis, and tetralogy of fallot had F1 scores > 0.90.
Zuercher et al. [53]	Automated function quantification	Deep learning—EchoNet-Dynamic	321 echocardiograms (267 normal, 54 with dilated cardiomyopathy)	Calculated LVEF with mean average error 4.47%. Bias of −2.42% compared to expert.

**Table 3:** Current Status, Challenges and Future Directions of AI in Fetal and Pediatric Echocardiography.

Category	AI Application	Challenges	Future Directions
Image Acquisition & Classification	- SonoNet [26,27] and FINE [25] improve view acquisition and accuracy - FINE reduces manual annotation and generates standardized views	- AI struggles with some complex views (e.g., ductal arch, SVC/IVC) - Variability in operator skill and fetal motion	- Further refinement to improve accuracy of complex or rare views - Development of adaptive AI to handle motion artifacts and low-quality images
Image Segmentation	- DW-Net [37] and SONO [23] used for automatic segmentation of fetal cardiac structures with 80% accuracy	- Difficulty in detecting small structures (e.g., mitral valve, IVC, pulmonary veins) - Small datasets for fetal segmentation	- Expand fetal echocardiography datasets - Improve segmentation of small and subtle cardiac structures, particularly in complex CHD
CHD Detection (Fetal Echocardiography)	Truong et al.’s random forest models [24] and Arnaout et al.’s CNNs [22] accurately detect CHD with AUCs > 0.90	- AI may miss subtle or rare CHD cases - Performance varies across different populations and settings	- Improve AI models to detect rare and subtle CHD cases - Create large, diverse, multi-center datasets for better generalizability across populations
Pediatric View Classification	Gearhart et al.’s AI model achieves high accuracy (>90%) in classifying standard views in pediatric echocardiography [46]	- Some views (e.g., subcostal short-axis sweep and parasternal views) have lower accuracy, especially in CHD patients	- Improve classification accuracy in complex cases and less commonly acquired views - AI model refinement for pediatric CHD view classification
Disease-specific Diagnosis	Lin et al.’s DL model diagnoses atrial septal defects with high sensitivity and specificity (AUC 0.92) [48]	- Struggles to generalize across different types of CHD - Diagnostic limitations for complex CHD	- Further develop AI to identify a broader spectrum of CHD types. - Improve AI algorithm to diagnose complex and mixed CHDs.
Automated Function Quantification	EchoNet-Peds and EchoNet-Dynamic retrained to calculate ejection fraction (mean error 3.66%) and cardiac output (R2 of 0.755, comparable to expert reviewers) [50,53]	- Limited datasets for model training - Higher error rates in complex CHD	- Expand pediatric-specific data to improve accuracy in measurements - Tailored AI tools for complex CHD to improve measurement accuracy
Clustering Analysis and Outcome Prognostication	Unsupervised cluster analysis identifies distinct CHD phenotypes and association of clinical features with outcomes in dilated cardiomyopathy and CHD [67]	- Lack of longitudinal data to train AI models for long-term outcome prediction - Difficulty integrating cross-institutional data	- Develop AI models for long-term risk prediction and patient outcomes - Collaborate across centers to create longitudinal datasets for training
Screening for Acquired Disease in Resource-Limited Settings	Brown et al.’s ML algorithm detected mitral regurgitation, improving identification of rheumatic heart disease (AUC 0.93) [43]	- Limited access to AI-equipped devices in resource-poor regions - Technical support and infrastructure are lacking in resource-poor regions	- Portable and accessible AI tools for handheld devices - Focus on increasing access to AI-guided echocardiography in low-resource settings
Education and Training	Used in education and simulation for training sonographers and cardiologists	- Limited exposure to AI technologies among clinicians and sonographers - Resistance to AI integration into clinical workflows	- Expand training programs and workshops on AI in echocardiography - Promote interdisciplinary collaboration for AI development and adoption

AI, artificial intelligence; AUC, area under the curve; CHD, congenital heart disease; DL, deep learning; FINE, Fetal Intelligent Navigation Echocardiography; IVC, inferior vena cava; SONO, Supervised Object detection with Normal data Only; SVC, superior vena cava.
